# Relationship between periodontal disease and butyric acid produced by periodontopathic bacteria

**DOI:** 10.1186/s41232-018-0081-x

**Published:** 2018-12-17

**Authors:** Michihiro Shirasugi, Maki Nakagawa, Keisuke Nishioka, Toshiro Yamamoto, Takaaki Nakaya, Narisato Kanamura

**Affiliations:** 1 Department of Infectious Diseases, Kyoto Prefectural University of Medicine, 465 Kajii-cho, Kawaramachi-Hirokoji, Kamigyo-ku, Kyoto, 602-8566 Japan; 2 Department of Dental Medicine, Kyoto Prefectural University of Medicine, 465 Kajii-cho, Kawaramachi-Hirokoji, Kamigyo-ku, Kyoto, 602-8566 Japan

**Keywords:** Periodontal diseases, Butyric acid, TNF-α, TNF-α receptor, HDAC inhibitor

## Abstract

**Background:**

Periodontopathic bacteria such as *Porphyromonas gingivalis* produce a large amount of butyric acid as a metabolite. Though butyric acid has been reported to have an anti-inflammatory effect on inflammatory diseases in the gastrointestinal tract, it has been suggested to contribute to the progression of periodontal disease in the oral cavity. The concentration of butyric acid in periodontal tissue of patients with periodontitis patients is reported to increase with the progress of the periodontal disease state. However, the influence of butyric acid on periodontal disease progression is not well known.

**Main text:**

In this review, we have considered the relationship between butyric acid and periodontal disease with respect to the findings reported till date and the knowledge we newly obtained [Shirasugi M et al. Biochem Biophys Res Commun, 2017]. We have studied the relationship between butyric acid and periodontal disease by analyzing the effect of butyric acid on normal human gingival fibroblasts, which are a major component of periodontal tissue. We observed that gingival fibroblasts underwent cytostasis and apoptosis via extrinsic and intrinsic pathways upon long-term exposure to butyric acid. In addition, we showed that TNF-α produced by gingival fibroblasts treated with butyric acid plays an important role in inducing exogenous apoptosis.

**Conclusion:**

Butyric acid produced by periodontopathic bacteria may promote progress of the periodontal disease state. Butyric acid is known to act as an HDAC inhibitor. Thus, we believe that advanced epigenetic analysis of the effects of butyric acid on gingival fibroblasts will help elucidate the periodontal disease pathology and facilitate discovery of new targets for periodontal disease treatment.

## Background

Periodontal disease is a lifestyle-related disease [1]. Periodontal disease often progresses without pain [2], and patients usually become aware of a periodontal disease only after appearance of symptoms such as gingival swelling, bleeding, pain, and tooth mobility. Progression of periodontal disease also leads to tooth loss. Eating and articulation functions are significantly hindered, and esthetic properties are deteriorated. Thus, periodontal disease leads to a marked decline in the quality of life [3]. Recently, periodontal disease has been reported to adversely affect the oral cavity as well as systemic diseases such as heart disease [4, 5], diabetes [6], and rheumatoid arthritis [7]. It has also been suggested that periodontal disease causes neurological disorders such as Alzheimer’s disease [8, 9]. Therefore, breakthroughs in treatment of periodontal disease are required more than ever. However, periodontal disease interacts complexly with pathogenic factors and reactions in the host, and so there are still many unknown points about its progression. Thus, it is important to clarify the detailed mechanism underlying periodontal disease progression to discover new targets of treatment. Past studies on periodontal diseases have been focused on pathogenic factors such as LPS from periodontal pathogens represented by the Red complex (*Porphyromonas gingivalis*, *Treponema denticola*, *Tannerella forsythensis*). However, little attention has been paid to the effect of metabolites produced by periodontal pathogens on the progression of periodontal diseases. The bacterial flora differs considerably at the supragingival and subgingival level and the metabolites produced are also different. The cariogenic bacteria in supragingival plaques, such as *Streptococcus mutans*, produce lactic acid, which induces dental caries by lowering the pH of dental enamel [10]. Periodontal pathogens such as *Porphyromonas gingivalis* and *Fusobacterium nucleatum* in subgingival plaques produce a large amount of short chain fatty acids including butyric acid as metabolites [11]. However, there are few reports on whether butyric acid contributes to the progression of periodontal diseases. In the gastrointestinal tract, butyric acid acts as an HDAC inhibitor and induces naive T cell differentiation into regulatory T cells by enhancing Foxp3 expression [12]. As a result, butyric acid has been suggested to exert an anti-inflammatory effect in gastrointestinal inflammatory diseases. In contrast, butyric acid is considered to act invasively on periodontal tissues [13–16]. Therefore, we aim to clarify the mechanism of periodontal disease progression and search for new therapeutic targets by investigating the effect of butyric acid on periodontal tissues in detail.

## Main text

### High concentration of butyrate-induced apoptosis in inflamed-human gingival fibroblasts

High concentrations of butyric acid are reported to induce apoptosis in immune cells [17–19]. This may result in the protection of butyrate-producing bacteria from phagocytosis by immune cells. It has also been reported that butyric acid promotes the activation of HIV and the onset of Kaposi’s sarcoma in the oral cavity [20]. Furthermore, inflammatory disease in the brain has been linked with increasing concentration of butyric acid in periodontal tissue [21]. Ochiai et al. have reported that high concentration of butyrate induces apoptosis in human gingival fibroblasts (HGFs) collected from inflamed sites of patients with periodontal disease [16]. This result is considered to be related to the frail periodontal tissue in periodontitis patients. On the other hand, they reported that high concentrations of butyric acid did not have a significant effect on healthy gingival fibroblasts. Responses to LPS from *Porphyromonas gingivalis* are known to differ between healthy and inflamed gingival fibroblasts [22]. HGFs collected from patients with periodontal diseases are less resistant to LPS from *Porphyromonas gingivalis* than HGFs collected from healthy subjects. The pro-inflammatory cytokine mRNA expression in inflamed subjects is reportedly upregulated by lower LPS and shorter stimulation time compared to that in healthy subjects [22]. Therefore, there is a possibility that healthy and inflamed gingival fibroblasts may show different reactions to butyric acid stimulation. In the above research [16], the exposure to butyric acid was up to 24 h. As periodontal disease is chronic in nature, we considered that periodontal tissues are exposed to pathogenic factors for a long time during periodontal disease progression. Furthermore, it is considered that normal (healthy) human gingival fibroblasts are also exposed to butyric acid for a long time in the process of periodontal disease progression. Therefore, we investigated and reported the effects of long-term exposure to butyric acid in normal HGFs, which constitute a major part of periodontal tissues [23].

### Long-term exposure of normal human gingival fibroblasts to butyric acid also induces apoptosis

Normal human gingival fibroblasts were reported to be less sensitive to butyric acid [16]. The number of viable normal HGFs was not changed after 24 h exposure to butyric acid; however, the viability of HGFs was significantly decreased compared with that of the control group (untreated) upon long-term exposure [16, 23]. We then estimated that the cause of decreased HGF viability was the suppression of cell division or induction of cell death by butyric acid stimulation. We first evaluated the suppression of cell division stimulated by butyric acid using flow cytometry with CFSE. At 24 h of exposure to butyric acid, no effect on cell division of HGFs was observed. However, after 24 h exposure to butyric acid, the cell division of HGFs was remarkably suppressed [23]. Subsequently, we examined the possibility that cell death was induced in HGFs by butyric acid. HGFs were stimulated by butyric acid and then stained with annexin V and PI [23]. We performed a cell death assay using flow cytometry and fluorescence microscopy. We thus observed that long-term exposure to butyric acid induced apoptosis in normal HGFs [23]. It has been reported that butyric acid induces apoptosis in T cells by activating Caspase 8 and Caspase 9 [19]. Therefore, further analysis of the apoptosis induction pathway revealed the activation of Caspase 8 and Caspase 9 in HGFs by butyric acid stimulation [23]. This indicated that HGFs underwent apoptosis via both the extrinsic and intrinsic pathways upon butyric acid stimulation. Furthermore, we found that the mRNA expression of Bak, a Bcl-2 family member that promoted cytochrome C release in mitochondria and induced intrinsic apoptosis, was enhanced by butyric acid in HGFs [23].

### Effect of butyric acid on pro-inflammatory cytokine production of HGFs

**Fig. 1:**
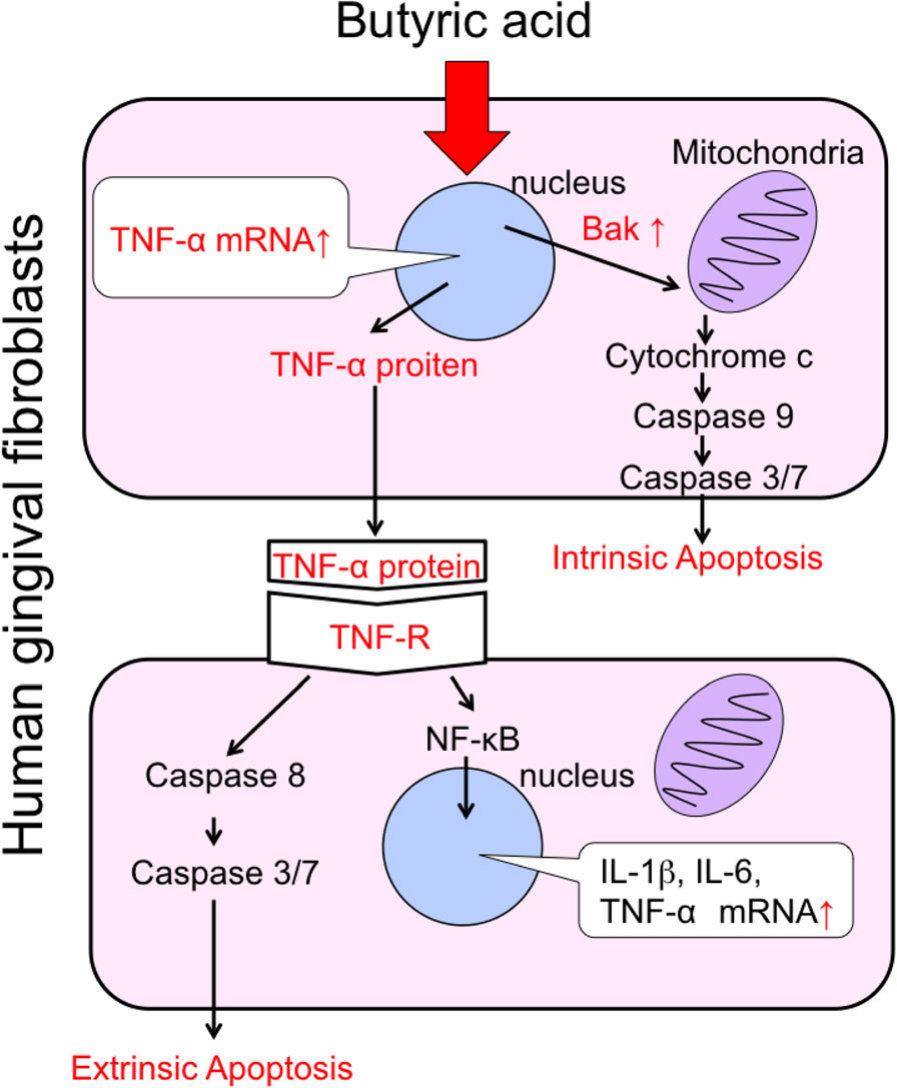
Intrinsic and extrinsic apoptosis pathway. Adapted and partially modified from [23]

Periodontal disease is also an inflammatory disease. The concentration of pro-inflammatory cytokines such as IL-6 and TNF-α in gingival crevicular fluid (GCF) increases with the progress of periodontal disease [24]. Pro-inflammatory cytokines such as IL-1β, IL-6, and TNF-α produced by monocytes, macrophages, and fibroblasts stimulated by pathogenic factors of periodontal pathogens contribute to the progress the periodontal disease as well as systemic diseases such as diabetes and rheumatoid arthritis [6, 7]. We therefore analyzed the effect of butyric acid on pro-inflammatory cytokine production in HGFs. We found that the mRNA expression of TNF-α, IL-1β, and IL-6 in HGFs were upregulated by butyric acid [23]. Among these, TNF-α mRNA was significantly upregulated at the early stage of butyric acid exposure [23]. Enzyme-linked immunosorbent assay revealed that HGFs produced TNF-α protein upon butyric acid stimulation [23]. We then hypothesized that this produced TNF-α might contribute to induce extrinsic apoptosis and upregulate the expression of pro-inflammatory cytokines. In order to demonstrate this assumption, HGFs were stimulated by butyric acid in the presence of TNF-α neutralizing antibody. In the presence of TNF-α neutralizing antibody, activation of Caspase 8 and expression of IL-6 mRNA were not observed in HGFs despite butyric acid stimulation [23]. These results suggest that TNF-α produced by HGFs exposed to butyric acid contributes to induce extrinsic apoptosis and enhance proinflammatory cytokine expression (Fig. 1). HGFs have been reported to rescue T cells from butyric acid stimulation [25]. It is conceivable that this defense reaction is countered by apoptosis induction in the gingival fibroblasts. From the above results, we consider that butyric acid produced by periodontal pathogens results in the collapse of periodontal tissue homeostasis and might promote progress of the periodontal disease state.

### Need for epigenetic analysis on the effect of butyric acid on HGFs

**Fig. 2:**
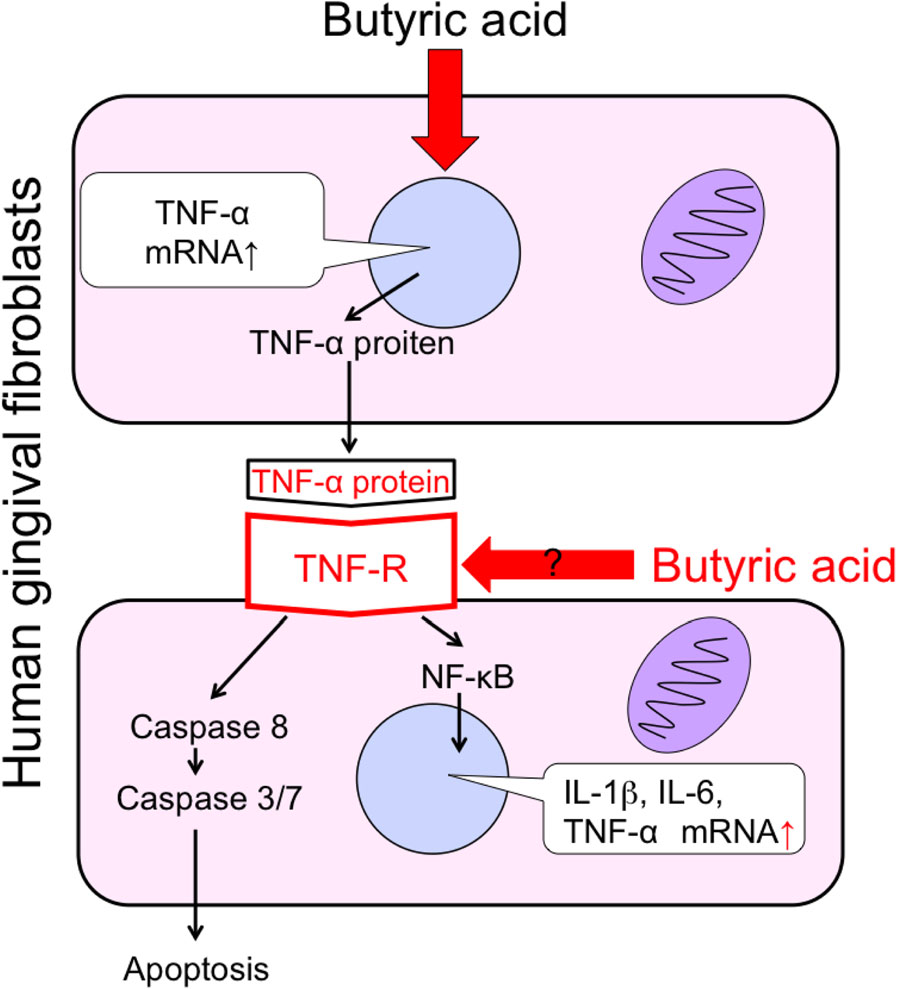
Butyric acid may alter the TNF-R of HGFs. Adopted and partially modified from [23]

Butyric acid is known to act as an HDAC inhibitor [12, 26]. HDAC inhibitors inhibit histone deacetylation and promote gene expression by facilitating the binding of transcription factors and RNA polymerase to DNA strands. In the gastrointestinal tract, butyric acid as HDAC inhibitor enhances Foxp3 expression in naive T cells and induces their differentiation into regulatory T cells [12]. In the oral cavity, butyric acid produced by the periodontal pathogen acts as an HDAC inhibitor and activates latent HIV [26]. For the same HDAC inhibitor, the site of action varies depending on the type of cells. Therefore, it is essential to perform epigenetic analysis on the effect of butyric acid on HGFs. As described above, it is likely that TNF-α plays an important role in the effect of butyric acid exposure to HGFs. Although the amount of TNF-α protein produced was extremely low, it exerted significant effects such as induction of extrinsic apoptosis and upregulation of proinflammatory cytokines in HGFs. Hence, the expression and susceptibility of the TNF-α receptor on HGFs might change upon butyric acid exposure (Fig. 2). In actual periodontitis pathology, immune cells migrate and produce large amounts of TNF-α [27]. Therefore, if the susceptibility of HGFs to TNF-α has been enhanced by butyric acid stimulation, there is a high possibility that the progression of periodontal disease pathology is promoted by butyric acid. Therefore, it is necessary to further investigate whether the susceptibility of HGFs to TNF-α is altered by butyric acid, using epigenetic analysis.

## Conclusions

Butyric acid produced by periodontal pathogens may collapse the homeostasis in periodontal tissues. Considering the metabolites of periodontal pathogens, which have not been noticed so far, may lead to the discovery of novel targets for treatment. It is thus important to further clarify the effect of butyric acid on periodontal tissues in order to elucidate the mechanism of progression of periodontal disease pathology and develop breakthrough treatments.

## Abbreviations

CFSE: Carboxyfluorescein diacetate succinimidyl ester
HDAC: Histone deacetylase
HGFs: Human gingival fibroblasts
